# Smoking and mortality after breast cancer diagnosis: the health and functioning in women study

**DOI:** 10.1002/cam4.359

**Published:** 2014-12-16

**Authors:** Monika Izano, William A Satariano, Robert A Hiatt, Dejana Braithwaite

**Affiliations:** 1 Department of Epidemiology and Biostatistics, University of CaliforniaSan Francisco, California; 2 School of Public Health, University of CaliforniaBerkeley, California; 3 Helen Diller Family Comprehensive Cancer Center, University of CaliforniaSan Francisco, California

**Keywords:** Breast cancer, Cohort study, mortality, smoking, survival

## Abstract

We examined the effect of smoking on long-term mortality from breast cancer and other causes among a cohort of women with breast cancer. A total of 975 women diagnosed with breast cancer and aged 40–84 years were followed for a median follow-up of 11 years in the U.S. Health and Functioning in Women (HFW) study. The impact of the individual smoking status and smoking intensity reported in the first few months following breast cancer diagnosis on the risk of mortality from breast cancer and other causes was examined using Cox proportional hazards models. In this study, former smoking was associated with increased risk of other-cause mortality (hazard ratio [HR] = 1.47, 95% confidence interval [CI]: 1.13–1.90), and the risk doubled with increased intensity (HR for <50 pack-years [py]: 1.36, 95% CI: 1.03–1.79; HR for ≥50 py: 2.45, 95% CI: 1.41–4.23). Current smoking (HR = 2.45, 95% CI: 1.81–3.32) and each additional 10 py smoked (HR = 1.16, 95% CI: 1.11–1.22) were associated with statistically significant increases in the risk of other-cause mortality. The effect of current smoking on other-cause mortality decreased with advancing stage and increasing body mass index (BMI). Breast cancer-specific mortality was associated with current smoking of ≥50 py (HR = 2.36, 95% CI: 1.26–4.44), and each additional 10 py smoked (HR = 1.07, 95% CI: 1.01–1. 14). Current smoking, but not former smoking, was associated with increased risk of breast cancer-specific mortality in women with local disease (HR = 2.32, 95% CI: 1.32–4.09), but not in those with regional and distant disease (HR = 1.10, 95% CI: 0.73–1.68). Our findings suggest that current smoking at the time of breast cancer diagnosis may be associated with increased risk of breast-cancer specific and other-cause mortality, whereas former smoking is associated with increased risk of other-cause mortality. Smoking cessation at the time of diagnosis may lead to better prognosis among women with breast cancer.

## Introduction

The identification of modifiable lifestyle factors that may improve survival is of interest to women diagnosed with breast cancer, as well as their caregivers. A mouse model of mammary cancer-linked cigarette smoke exposure to an increase in the total pulmonary metastatic burden 1, and epidemiologic studies have linked smoking among breast cancer survivors to adverse prognostic factors, such as more and larger lymph node metastasis 2,3 and increased risk of developing metastasis to the lung 1,4. It is therefore plausible that smoking could increase the risk of breast cancer death. Smoking has been associated with higher mortality following diagnosis of breast 5,6, prostate 7,8, colorectal 9, and vulvar 10 cancers, as well as leukemia 11 and malignant melanoma 12. Yet, the association of smoking with breast cancer-specific mortality appears equivocal 6,13–18. The majority of studies of smoking and breast cancer survival have used crude measures of smoking history, such as never, former, and current smoking 6,13,15,17–19 or ever and never smoking 14. Two studies additionally evaluated the number of cigarettes smoked per day among current smokers 6,14, and one of these also considered the number of years smoked and the age at which respondents started smoking 14. A third study additionally considered the number of pack-years (py) smoked 17. To date, the associations between measures of smoking exposure length, intensity, and smoking cessation with breast cancer-specific mortality have not been evaluated.

Based on epidemiological and biological evidence that smoking may influence breast cancer progression, we hypothesized that smoking would be associated with both increased risk of death from breast cancer, as well as death from other causes in our prospective study of 975 African–American and white breast cancer survivors from the Health and Functioning in Women (HFW) study 20. Comprehensive measures of smoking history, a wide age range, the inclusion of both African–American and white women and a maximum follow-up of 28 years make this cohort particularly suitable for examining the long-term effects of smoking while taking into account a wide range of clinical, lifestyle-related, and socio-demographic prognostic factors. Since smoking can disrupt hormonal metabolism, the effect of smoking might differ by body mass index (BMI). Furthermore, although smoking has been associated with adverse prognostic factors, such as more and larger lymph node metastasis 2,3 and increased risk of developing metastasis to the lung 1,4, the extent to which disease severity affects the impact of smoking on mortality is not well understood. Therefore, we also examined the extent to which the impact of smoking on mortality differed as a function of tumor stage and BMI.

## Methods

### Study population

The HFW study used in the present analysis has been described previously 20,21. Briefly, the HFW study was established in 1984 in the Detroit metropolitan area to assess the health, functional and psychosocial status of women following breast cancer diagnosis. A total of 1011 eligible participants aged 40–84 with newly diagnosed, histologically confirmed, primary invasive breast cancer identified through the Metropolitan Detroit Cancer Surveillance System (MDCSS) at the Michigan Cancer Foundation, now the Barbara Ann Karmanos Cancer Institute, within 4 weeks of diagnosis and were interviewed in two cohorts. The registry data were over 90% complete. The first cohort consisted of 571 participants aged 55–84, who were identified over a 7-month period between 1984 and 1985; of these, 463 (81.1%) were successfully interviewed between 2 and 4 months following diagnosis. A second cohort of 620 eligible cases, aged 40–54 and 74–84, was identified over a 7-month period between 1987 and 1988; 548 (88.4%) of these participants were successfully interviewed between 2 and 4 months after diagnosis, henceforth referred to as the baseline interview. All participants were interviewed a second time approximately 9 months after the first interview. The two cohorts were combined, and 975 women, for whom complete data were available on smoking and breast cancer staging, were included in this analysis.

### Smoking assessment

Smoking status was determined from the baseline questionnaire after breast cancer diagnosis. The questionnaire asked whether the respondent ever smoked cigarettes daily. Never smokers were women who answered “no” to this question. Women were also asked if they smoked at the time of the interview. Respondents that answered “yes” to this question were considered current smokers. Women that answered “yes” to ever having smoked and “no” to the current smoking question were considered former smokers. Smoking status, one of the exposures we considered, was created as a three-level variable indicating whether the respondent was a never/former/or current smoker. Additionally, current and former smokers were asked whether, when they smoked daily, they smoked less than 0.5 packs (1 pack = 20 cigarettes), 0.5 packs, 1 pack, 1.5 packs, or two packs or more a day and the total number of years during which they smoked. The number of py was computed as the product of the packs per day and the number of years smoked. The number of pack-years was used as a continuous predictor in the models. Effect estimates were reported in terms of 10 py increments. The number of “less than 0.5 packs per day” was treated as 0.5 packs, and two packs or more were conservatively counted as two packs. A third exposure combined the number of pack-years and smoking status into a five-level categorical predictor of both longevity and intensity of smoking (never smoked, former smoker having smoked <50 py, former smoker having smoked ≥50 py, current smoker having smoked <50 py, and current smoker having smoked ≥50 py), because this measure was shown to predict mortality in middle-aged women of the general population 22. In sensitivity analyses, instead of 50 py, a 20 py cutoff was used to define exposure categories. Results from these analyses are provided in Table S1.

### Covariates

The covariates used in this analysis were socio-demographic, lifestyle-related, and clinical prognostic factors that, based on the existing literature and a priori reasoning, could potentially confound associations between smoking and mortality outcomes. Age at diagnosis, breast cancer stage, breast cancer treatment, tumor size, and node involvement were obtained from the MDCSS file, while other variables were obtained from interviews. In analyses, age was used as a continuous variable. Race was coded as either African–American or white. Years of education were recoded into four categories: less than high-school, high-school, college and graduate. The data set included a binary indicator of financial adequacy (0 for adequate and 1 for inadequate) that was based on self-reported current financial resources and whether they met the participant's needs 21. BMI was calculated as weight in kilograms/height in meters squared, or kg/m^2^, from self-reported weight and height at the baseline interview and used as the continuous variable in multivariate models. A comorbidity index was constructed as the number of previously diagnosed conditions reported by the respondents at the baseline interview from a list of 23 conditions that included diabetes, hypertension, stroke, heart disease, gastrointestinal disease, liver conditions, and primary cancers other than breast cancer, which, according to the respondents, currently caused some limitations in their activities 21.

Stage of breast cancer at diagnosis was coded as local, regional, or remote. In addition to information on surgery (no surgery, partial mastectomy, or modified radical mastectomy) and type of adjuvant therapy (radiation or chemotherapy) provided by the MDCSS files, physicians completed a supplementary survey regarding chemotherapy and hormonal therapy administered on an outpatient basis. Data from the two sources were combined to create a two-level treatment variable (no surgery or partial mastectomy and modified radical mastectomy). The log of the tumor size in millimeters was centered on its mean and used as a continuous variable. The number of positive lymph nodes involved was recoded into a three-level variable (0 nodes, 1–3 nodes and ≥4 nodes).

### Endpoint ascertainment

Participants were followed until last contact, death or April, 2012, whichever occurred first. Date and cause of death, classified by the International Classification of Diseases (ICD) codes version 9, were identified through annual vital status surveillance of all patients in the registry, conducted by MDCSS 23. ICD codes 174.0–174.9 represented breast cancer deaths, and other ICD codes represented death from causes other than breast cancer, referred to henceforward as other-cause mortality. Vital status assessment was complete for all participants, and the cause of death was available for all participants that died.

### Statistical analysis

Differences between continuous variables were assessed using the Student's *t*-test, and between categorical variables were assessed by Pearson *χ*^2^ or by Fisher's exact test when counts were small. Differences in sample medians were assessed using the Wilcoxon Rank Sum test. Kaplan–Meier plots were used to examine the association between smoking and mortality. Cox proportional hazards models stratified by age at breast cancer diagnosis with time since diagnosis as the time scale were employed to estimate the association between smoking and other-cause and breast cancer mortality 24. Risk was expressed as a hazard ratio (HR) and 95% confidence interval (CI). The proportionality of hazards assumption was assessed using Schoenfeld residuals 25. These tests revealed no significant departures from proportionality. For analyses involving death from breast cancer, participants who died from other causes were censored at the time of their death and vice versa. Comorbidity, treatment, tumor stage, tumor size, node involvement, race, BMI, financial adequacy, education, smoking status and period of study entry were considered as potential confounders in all multivariate analyses. To evaluate effect modification, we conducted analyses separately for subgroups defined by BMI (<25, 25–30, >30) at the baseline interview and stage of breast cancer (local, regional or distant) at diagnosis. We combined women in the regional and distant categories due to the small number of respondents with distant disease (*n* = 55). The type I error was set at 0.05, and all reported *P*-values are two-sided. Analyses were conducted in SAS version 9.2 (SAS Institute, Cary, NC) and R version 2.15.

## Results

The distributions of selected demographic and clinical characteristics of the study population, overall and by smoking status, are presented in Table1. The mean age at the time of breast cancer diagnosis was 63 years (SD [standard deviation] = 12.4). On average, current and former smokers were younger than never smokers (*P* < 0.0001). Overall, the median follow-up time was 11.0 years (interquartile range [IQ]: 4.5–22.4 years). The median follow-up was significantly shorter for current smokers than former and never smokers (12, 11, and 6 years for never smokers, former smokers ≥50 py, and current smokers ≥50 py, respectively; *P *= 0.004). There were statistically significant differences in BMI by smoking status. Mean BMI was 26.2 (SD = 4.7), 28.5 (SD = 5.0), and 24.8 (SD = 5.6) kg/m^2^ among never, former ≥50 py and current smokers ≥50 py, respectively (*P* = 0.004). Never and former smokers tended to be more educated than current smokers (*P* = 0.004). Breast cancer staging did not differ by smoking status. The majority of women had localized (53.6%) and regional (40.7%) disease. Only 5.6% of the group had remote disease. Treatment also did not vary significantly by smoking status: 78% of women were treated with modified radical mastectomy; 20% were treated with partial mastectomy; and only 2% received nonsurgical treatment. Of note, among women that died during the follow-up, current and former smokers were proportionately more likely to die from breast cancer compared to never smokers. Approximately 46% of former and current smoker deaths were due to breast cancer compared to 39% of deaths among the never smokers. Tumor size, lymph node involvement, and the number of comorbidities did not vary significantly by smoking status.

**Table 1:** Characteristics of the study group overall and by smoking status

	Overall (*N* = 975)	Never (*N* = 494)	Former <50 pack-years (*N* = 251)	Former ≥50 pack-years (*N* = 35)	Current <50 pack-years (*N* = 164)	Current ≥50 pack-years (*N* = 31)	*P*-value
Age at diagnosis, mean ± SD	63 ± 12.4	66.1 ± 12.3	60.3 ± 12.4	61.5 ± 11.6	57 ± 9.7	68 ± 10	<0.0001
Follow-up (years), median (*Q*1, *Q*3)	11 (4.5, 22.4)	12 (4.5, 21.3)	14 (4.4, 24.1)	11 (6.1, 23)	12 (4.1, 22.6)	6 (3.1, 9.1)	0.004
Financial adequacy 1, *n* (%)	807 (82.8)	408 (82.6)	211 (84.1)	28 (80)	132 (80.5)	28 (90.3)	0.67
Highest level of educational attainment, *n* (%)							
Less than high-school	375 (38.5)	213 (43.1)	82 (32.7)	12 (34.3)	55 (33.5)	13 (41.9)	0.004
High-school	330 (33.8)	154 (31.2)	82 (32.7)	16 (45.7)	67 (40.9)	11 (35.5)	
College	210 (21.5)	98 (19.8)	64 (25.5)	5 (14.3)	36 (22)	7 (22.6)	
Graduate	60 (6.2)	29 (5.9)	23 (9.2)	2 (5.7)	6 (3.7)	0 (0)	
Body mass index (kg/m^2^), mean ± sd	26.3 ± 5.3	26.2 ± 4.7	26.9 ± 5.7	28.5 ± 5	25.5 ± 5.9	24.8 ± 5.6	0.004
Stage, *n* (%)							
Local	523 (53.6)	278 (56.3)	126 (50.2)	20 (57.1)	83 (50.6)	16 (51.6)	0.49
Regional	397 (40.7)	186 (37.7)	107 (42.6)	14 (40)	77 (47)	13 (41.9)	0.29
Distant	55 (5.6)	30 (6.1)	18 (7.2)	1 (2.9)	4 (2.4)	2 (6.5)	0.24
Breast cancer treatment, *n* (%)							
No surgery	17 (1.7)	13 (2.6)	4 (1.6)	0 (0)	0 (0)	0 (0)	0.17
Partial mastectomy	194 (19.9)	95 (19.2)	54 (21.5)	12 (34.3)	28 (17.1)	5 (16.1)	0.18
Modified radical mastectomy	760 (77.9)	384 (77.7)	191 (76.1)	23 (65.7)	136 (82.9)	26 (83.9)	0.16
Number of lymph nodes involved, *n* (%)							
0	443 (45.4)	241 (48.8)	107 (42.6)	16 (45.7)	63 (38.4)	16 (51.6)	0.22
1–3	302 (31)	137 (27.7)	83 (33.1)	9 (25.7)	61 (37.2)	12 (38.7)	
≥4	39 (4)	22 (4.5)	11 (4.4)	2 (5.7)	4 (2.4)	0 (0)	
Tumor size (mm), mean ± SD	33.7 ± 25	32.9 ± 24.3	36.9 ± 27	31.2 ± 23.8	32 ± 24.3	31.4 ± 24.4	0.22
Number of comorbidities, mean ± sd	2.2 ± 1.5	2.2 ± 1.5	2.1 ± 1.4	2.7 ± 1.9	2.2 ± 1.3	2.5 ± 1.7	0.13
Mortality*, n* (%)							
All-cause	753 (77.3)	389 (78.7)	179 (71.3)	27 (77.1)	129 (78.7)	29 (96.7)	0.02
Breast cancer-specific 2	317 (42.1)	151 (38.8)	84 (46.9)	10 (37.0)	59 (45.7)	13 (44.8)	0.50
Other-cause 2	436 (57.9)	238 (61.2)	95 (53.1)	17 (63.0)	70 (54.3)	16 (55.2)	0.08

1 A binary indicator of whether the participant's current financial resources met their needs (yes/no).

2 The total number of deaths is used as the denominator.

### Breast cancer mortality

**Figure 1:**
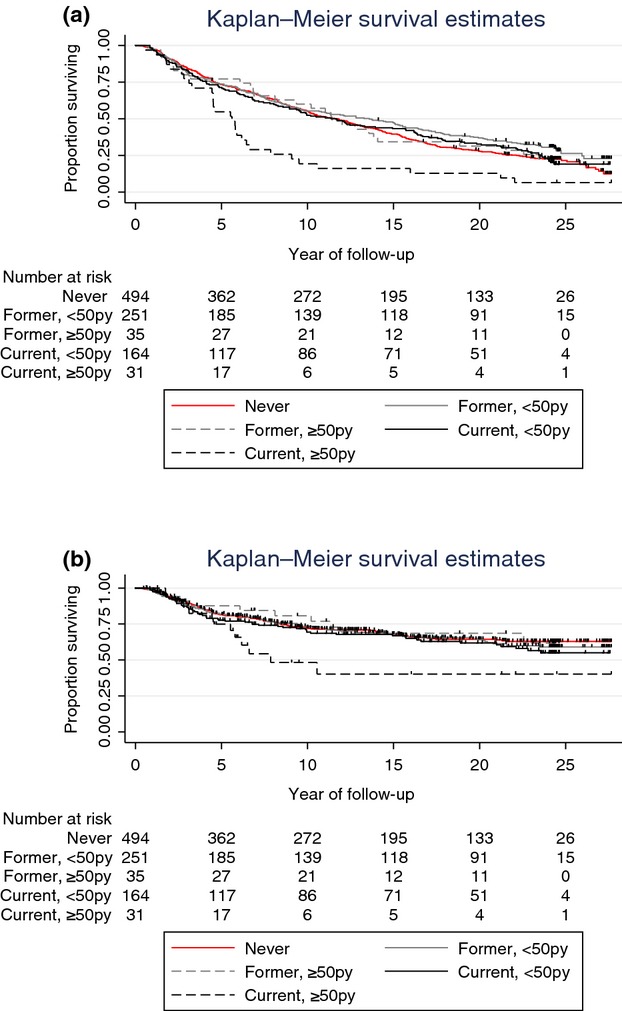
(A) Kaplan–Meier estimates of other-cause survival by smoking category. (B) Kaplan–Meier estimates of breast cancer-specific survival by smoking category.

Kaplan–Meier plots of breast cancer-specific survival by smoking status indicate markedly shorter survival for current smokers of more than 50 py and similar survival among the remaining groups (Fig.1B). In univariate and covariate-adjusted analyses alike, we found no evidence of an association between smoking status and breast cancer-specific mortality (Table2). In analyses of smoking intensity, current smoking of ≥50 py was associated with a twofold increase in the risk of breast cancer-specific mortality in covariate-adjusted models (HR = 2.36, 95% CI: 1.26–4.44). In sensitivity analyses, current smoking of more than ≥20 py was also associated with a statistically significant increase in the risk of breast cancer-specific mortality (HR = 1.57, 95% CI: 1.10–2.25, Table S1). Each additional 10 py smoked was associated with a modest, but statistically significant increase in breast-specific mortality in covariate-adjusted models (HR = 1.07, 95% CI: 1.01–1.14). While the effect of smoking on breast cancer mortality did not vary significantly across strata defined by BMI (Table3), we found that among women with localized breast cancer, current smoking was associated with an approximately twofold increased risk of breast cancer death (HR = 2.32; 95% CI: 1.32–4.09) compared to never smoking in covariate-adjusted models. Current and former smoking were not associated with breast cancer-specific mortality in women with regional and distant breast cancer stage at the time of diagnosis.

**Table 2:** Hazard ratios (and 95% confidence intervals) of smoking for mortality

	*N*	Other-cause mortality (no. deaths = 436)			Breast cancer mortality (no. deaths = 317)		
Exposure		Deaths	Unadjusted HR (95% CI)	Adjusted HR (95% CI)	Deaths	Unadjusted HR (95% CI)	Adjusted HR (95% CI)
Smoking status							
Never smoker	494	238	1	1	151	1	1
Former smoker	286	112	1.42 (1.10, 1.83)	1.47 (1.13, 1.90)	94	1.06 (0.81, 1.40)	0.94 (0.70, 1.26)
Current smoker	195	86	2.51 (1.87, 3.37)	2.45 (1.81, 3.32)	72	1.33 (0.98, 1.80)	1.38 (0.99, 1.91)
Smoking status and intensity							
Never smoker	494	238	1	1	151	1	1
Former smoker, <50 py	251	95	1.32 (1.01, 1.73)	1.36 (1.03, 1.79)	84	1.08 (0.81, 1.43)	0.93 (0.69, 1.26)
Former smoker, ≥50 py	35	17	2.26 (1.33, 3.84)	2.45 (1.41, 4.23)	10	0.91 (0.47, 1.75)	0.91 (0.46, 1.81)
Current smoker, <50 py	164	70	2.54 (1.85, 3.50)	2.44 (1.75, 3.39)	59	1.22 (0.88, 1.69)	1.24 (0.87, 1.76)
Current smoker, ≥50 py	31	16	2.43 (1.36, 4.33)	2.55 (1.41, 4.62)	13	2.11 (1.15, 3.89)	2.36 (1.26, 4.44)
10 Pack-years	975	436	1.15 (1.10, 1.21)	1.16 (1.11, 1.22)	317	1.07 (1.01, 1.13)	1.07 (1.01, 1.14)

Unadjusted models were stratified by age at breast cancer diagnosis. Multivariate models were additionally adjusted for breast cancer treatment, race/ethnicity, body mass index, financial adequacy, education, positive lymph node involvement, tumor size at diagnosis, comorbidity, and period of study entry. py, pack-years.

**Table 3:** Hazard ratios (and 95% confidence intervals) of smoking status for mortality stratified by tumor stage and body mass index (BMI)

		Other-cause mortality (no. deaths = 436)			Breast cancer mortality (no. deaths = 317)		
	*N*	Deaths	Unadjusted HR (95% CI)	Adjusted HR (95% CI)	Deaths	Unadjusted HR (95% CI)	Adjusted HR (95% CI)
Stage of breast cancer at diagnosis^1^							
* * Local							
Never smoker	278	157	1	1	42	1	1
Former smoker	146	67	1.26 (0.90, 1.75)	1.30 (0.92, 1.83)	25	1.04 (0.61, 1.75)	0.90 (0.52, 1.56)
Current smoker	99	50	3.33 (2.24, 4.95)	3.18 (2.11, 4.81)	26	1.82 (1.07, 3.11)	2.32 (1.32, 4.09)
Regional and distant							
Never smoker	216	81	1	1	109	1	1
Former smoker	140	45	1.99 (1.22, 3.27)	1.91 (1.14, 3.19)	69	0.94 (0.66, 1.33)	0.91 (0.64, 1.31)
Current smoker	96	36	1.88 (1.10, 3.21)	1.87 (1.06, 3.30)	46	1.01 (0.67, 1.50)	1.10 (0.73, 1.68)
Body mass index at baseline, (kg/m^2^)^2^							
<25							
Never smoker	224	110	1	1	55	1	1
Former smoker	119	51	1.60 (1.07, 2.39)	1.59 (1.03, 2.45)	36	1.31 (0.82, 2.08)	1.18 (0.72, 1.95)
Current smoker	111	49	2.58 (1.61, 4.14)	2.83 (1.71, 4.68)	37	1.56 (0.96, 2.56)	1.34 (0.78, 2.29)
25–30							
Never smoker	177	87	1	1	61	1	1
Former smoker	98	39	1.42 (1.10, 1.83)	1.46 (1.13, 1.90)	30	1.06 (0.81, 1.40)	0.95 (0.71, 1.28)
Current smoker	48	22	2.51 (1.87, 3.37)	2.49 (1.84, 3.36)	18	1.33 (0.98, 1.80)	1.31 (0.95, 1.80)
>30							
Never smoker	93	41	1	1	35	1	1
Former smoker	69	22	1.70 (0.82, 3.54)	—	28	1.25 (0.66, 2.36)	0.90 (0.41, 1.97)
Current smoker	36	15	2.28 (0.90, 5.78)	—	17	2.13 (0.99, 4.55)	2.09 (0.86, 5.08)

Unadjusted models were stratified by age at breast cancer diagnosis. Multivariate models were additionally adjusted for breast cancer treatment, race/ethnicity, financial adequacy, education, positive lymph node involvement, tumor size at diagnosis, comorbidity, and period of study entry. —, Could not be estimated due to a small number of cases.

1 Models additionally adjusted for body mass index.

2 Models additionally adjusted for stage at diagnosis.

### Mortality from causes other than breast cancer

Kaplan–Meier plots of other-cause survival indicate decreasing survival by increasing smoking intensity (Fig.1A), with current smokers of ≥50 py having markedly shorter survival than the remaining groups, throughout the follow-up. After the initial 10 years of follow-up, former smokers of less than 50 py experienced better survival then current and never smokers. Results from unadjusted and covariate-adjusted analyses are presented in Table2. Former and current smoking were associated with increased risk of other-cause mortality in univariate and covariate-adjusted models alike. The risk was higher among current smokers, with covariate-adjusted HR of 1.47 (95% CI: 1.13–1.90) and 2.45 (95% CI: 1.81–3.32) for former and current smoking, respectively. The risk doubled with increasing intensity among former smokers, with covariate-adjusted HR of 2.45 (95% CI: 1.41–4.23) and 1.36 (95% CI: 1.03–1.79) for those having smoked more than 50 py and less than 50 py, respectively. Intensity did not greatly affect risk among current smokers, with covariate-adjusted HR of 2.55 (95% CI: 1.41–4.62) and 2.44 (95%CI: 1.75–3.39) for current smokers having smoked more than 50 py and less than 50 py, respectively. Lowering the pack-year cutoff from 50 to 20 in sensitivity analyses did not appreciably change the results (Table S1). The risk of death from causes other than breast cancer also increased with every additional 10 py smoked (HR = 1.16, 95% CI: 1.11–1.22).

Possible variations in the effect of smoking status on other-cause mortality according to tumor stage and BMI at diagnosis were also evaluated. Current smoking was associated with increased risk of other-cause mortality in women with local and regional/distant disease alike, but the risk was greater among women with local disease. HR for other-cause mortality were 3.18 (95% CI: 2.11–4.81) and 1.87 (95% CI: 1.06–3.30) for women with local and regional/distant disease at diagnosis, respectively (Table3). Former smoking was associated with increased risk in women with regional/distant disease (HR = 1.91, 95% CI: 1.14–3.19), but not among those with localized tumors at diagnosis (HR = 1.30, 95% CI: 0.92–1.83). In analyses stratified by BMI, former smoking was associated with increased risk of other-cause mortality in normal (HR = 1.59, 95% CI: 1.03–2.45) and over-weight women (HR = 1.46, 95% CI: 1.13–1.90), but not among those that were obese (HR = 1.38, 95% CI: 0.60–3.16). Current smoking also resulted in increased risk of other-cause mortality among normal-weight (HR = 2.83, 95% CI: 1.71–4.68), and overweight (HR = 2.49, 95% CI: 1.84–3.36) women, but not among obese women (HR = 1.94, 95% CI: 0.67–5.65).

## Discussion

In this study, we evaluated the association of smoking, as reported 2–4 months after diagnosis, with breast cancer-specific and other-cause mortality among breast cancer survivors. We found that former smoking was associated with increased risk of other-cause mortality, and the risk doubled with increasing intensity. Current smoking and every additional 10 py smoked were also associated with statistically significant increases in the risk of other-cause mortality. The effect of current smoking on other-cause mortality decreased with advancing stage and increasing BMI. Breast cancer-specific mortality was associated with current smoking of ≥50 py and each unit increase in 10 py smoked. Current smoking was associated with increased risk of breast cancer-specific mortality in women with local disease, but not in those with regional and distant disease. We found no evidence that smoking reduced the risk of breast cancer mortality by promoting lower body weight.

Reports of the association between smoking and breast cancer-specific mortality have been mixed. While some studies were able to establish a statistically significant association 18,19,26–29, others did not 6,15,30–33. Similar to other studies, using only smoking status following breast cancer diagnosis, we found that current and former smoking were associated with other-cause mortality, but not breast cancer mortality. The risk associated with current smoking was double that associated with former smoking, indicating that, most likely, current smoking is a surrogate for high lifetime exposure. Among former smokers, cumulative lifetime exposure of more than 50 py resulted in similar risk as current smoking of the same duration. These results are consistent with those reported by a pooled sample of 9975 breast cancer survivors from the Women's Health Eating and Living (WHEL) Study, the Life After Cancer Epidemiology (LACE) Study and the Nurses' Health Study (NHS) 29. These data indicate that epidemiologic studies of smoking, as well as those considering smoking as a potential confounder, should consider lifetime smoking exposure in addition to current smoking status.

The impacts of smoking on cardiovascular and respiratory health that lead to premature death are well documented. The biological pathways linking smoking to breast cancer-specific mortality are, on the other hand, less clear. Since breast cancer is a highly heterogeneous disease 34, it is plausible that smoking differentially affects estrogen-responsive tumors *versus* more aggressive forms of this cancer. In addition, cigarette smoke has been associated with the elevated metastatic potential of tumor cells and stimulation of angiogenesis 35. The association of cigarette smoke with an increased pulmonary metastatic propensity in mouse models 1 gives rise to the hypothesis that smoking could increase the risk of breast cancer recurrence and death. Consistent with this, cross-sectional epidemiologic evidence has shown that smokers with breast cancer had more and larger lymph node metastases than nonsmokers, after controlling for primary tumor size and other variables 2,3. In addition, smoking has been associated with a younger age at diagnosis 36, hormone receptor-negative breast cancer 37 and an increased risk of lung metastases among breast cancer patients 1,4. Although the mechanisms that lead to the altered tumor behavior associated with smoking are not well established, it has been shown that some complications of radiation therapy may be more frequent and severe in smokers 38. Notably, never smokers were more likely to receive chemotherapy than current or former smokers. Analysis of smoking status among women undergoing breast cancer treatment may help better target therapeutic options.

Limitations of this study include the fact that smoking status, duration, and intensity were self-reported and only once at the baseline examination after diagnosis. Since breast cancer is a disease with constant attrition for up to 30 years, it is possible that additional effects of smoking or more precision may have been observed with a longer follow-up. Another limitation is that smoking was assessed retrospectively, 2–4 months after breast cancer diagnosis. Given the considerable decline in smoking over the past 30 years in the United States 39, these data may not reflect contemporary patterns of exposure. Since smoking intensity and duration may not be collinear 40, pack-years may not be the best unit of exposure assessment. However, since age is associated with intensity and the duration of smoking is a function of age, it can be expected that intensity and duration will be collinear in the U.S. population of the recent decades 28. In addition, the number of participants in some of the highest intensity categories was relatively small. Another important limitation of this study is that the majority of respondents were treated with partial or radical mastectomy, which is no longer the standard of care for early-stage breast cancer. The currently recommended standard of care for this population consisting of radiation and segmental mastectomy or lumpectomy may have less impact on other-cause survival. Finally, the current study could not assess whether the impact of smoking on other-cause mortality differs in women with and without breast cancer. We were, however, able to compare smoking history among breast cancer cases and healthy controls and found no considerable differences between the two groups.

The strengths of the study include comprehensive measures of smoking status, as well as a composite measure of duration and intensity, a prospective population-based cohort design, a relatively large set of participants, a long follow-up and our ability to take into account multiple covariates in the tumor-related, lifestyle, and socio-demographic domains. Since women in this study were identified through a large regional, population-based surveillance program, our findings may apply to wider audiences than studies in which subjects were drawn from academic settings. Bias due to loss to follow-up was minimized because mortality status was ascertained annually for all of the patients in the registry.

In summary, our findings indicate that cigarette smoking confers a dose-dependent increasing risk of breast cancer-specific and other-cause mortality for women with breast cancer.

## Supporting Information

**Table S1.** Hazard ratios (and 95% confidence intervals) of smoking intensity for mortality.

## References

[b1] Murin S, Inciardi J (2001). Cigarette smoking and the risk of pulmonary metastasis from breast cancer. Chest.

[b2] Daniell HW (1988). Increased lymph node metastases at mastectomy for breast cancer associated with host obesity, cigarette smoking, age, and large tumor size. Cancer.

[b3] Daniell HW, Tam E, Filice A (1993). Larger axillary metastases in obese women and smokers with breast cancer–an influence by host factors on early tumor behavior. Breast Cancer Res. Treat.

[b4] Scanlon EF, Suh O, Murthy SM, Mettlin C, Reid SE, Cummings KM (1995). Influence of smoking on the development of lung metastases from breast cancer. Cancer.

[b5] Yu GP, Ostroff JS, Zhang ZF, Tang J, Schantz SP (1997). Smoking history and cancer patient survival: a hospital cancer registry study. Cancer Detect. Prev.

[b6] Holmes MD, Murin S, Chen WY, Kroenke CH, Spiegelman D, Colditz GA (2007). Smoking and survival after breast cancer diagnosis. Int. J. Cancer.

[b7] Daniell HW (1995). A worse prognosis for smokers with prostate cancer. J. Urol.

[b8] Myers RP (1995). Prostate cancer–neurovascular preservation; smoking cessation may enhance prognosis?. J. Urol.

[b9] Jadallah F, McCall JL, van Rij AM (1999). Recurrence and survival after potentially curative surgery for colorectal cancer. N. Z. Med. J.

[b10] Kirschner CV, Yordan EL, De Geest K, Wilbanks GD (1995). Smoking, obesity, and survival in squamous cell carcinoma of the vulva. Gynecol. Oncol.

[b11] Archimbaud E, Maupas J, Lecluze-Palazzolo C, Fiere D, Viala JJ (1989). Influence of cigarette smoking on the presentation and course of chronic myelogenous leukemia. Cancer.

[b12] Rigel DS, Friedman RJ, Levine J, Kopf AW, Levenstein M (1981). Cigarette smoking and malignant melanoma. Prognostic implications. J. Dermatol. Surg. Oncol.

[b13] Ewertz M, Gillanders S, Meyer L, Zedeler K (1991). Survival of breast cancer patients in relation to factors which affect the risk of developing breast cancer. Int. J. Cancer.

[b14] Calle EE, Miracle-McMahill HL, Thun MJ, Heath CW (1994). Cigarette smoking and risk of fatal breast cancer. Am. J. Epidemiol.

[b15] Hellmann SS, Thygesen LC, Tolstrup JS, Gronbaek M (2010). Modifiable risk factors and survival in women diagnosed with primary breast cancer: results from a prospective cohort study. Eur. J. Cancer Prev.

[b16] Warren GW, Kasza KA, Reid MA, Cummings KM, Marshall JR (2012). Smoking at diagnosis and survival in cancer patients. Int. J. Cancer.

[b17] Barnett GC, Shah M, Redman K, Easton DF, Ponder BA, Pharoah PD (2008). Risk factors for the incidence of breast cancer: do they affect survival from the disease?. J. Clin. Oncol.

[b18] Braithwaite D, Izano M, Moore DH, Kwan ML, Tammemagi MC, Hiatt RA (2012). Smoking and survival after breast cancer diagnosis: a prospective observational study and systematic review. Breast Cancer Res. Treat.

[b19] Warren GW, Kasza KA, Reid ME, Cummings KM, Marshall JR (2013). Smoking at diagnosis and survival in cancer patients. Int. J. Cancer.

[b20] Satariano WA, Ragheb NE, Branch LG, Swanson GM (1990). Difficulties in physical functioning reported by middle-aged and elderly women with breast cancer: a case-control comparison. J. Gerontol.

[b21] Satariano WA, Ragland DR (1996). Upper-body strength and breast cancer: a comparison of the effects of age and disease. J. Gerontol. A Biol. Sci. Med. Sci.

[b22] Tice JA, Kanaya A, Hue T, Rubin S, Buist DS, Lacroix A (2006). Risk factors for mortality in middle-aged women. Arch. Intern. Med.

[b23] Satariano WA, Ragland DR (1994). The effect of comorbidity on 3-year survival of women with primary breast cancer. Ann. Intern. Med.

[b24] Greenland S (1989). Modeling and variable selection in epidemiologic analysis. Am. J. Public Health.

[b25] Grambsch PM, Therneau TM (1994). Proportional hazards tests and diagnostics based on weighted residuals. Biometrika.

[b26] Tominaga K, Andow J, Koyama Y, Numao S, Kurokawa E, Ojima M (1998). Family environment, hobbies and habits as psychosocial predictors of survival for surgically treated patients with breast cancer. Jpn. J. Clin. Oncol.

[b27] Manjer J, Andersson I, Berglund G, Bondesson L, Garne JP, Janzon L (2000). Survival of women with breast cancer in relation to smoking. Eur. J. Surg.

[b28] Saquib N, Stefanick ML, Natarajan L, Pierce JP (2013). Mortality risk in former smokers with breast cancer: pack-years vs. smoking status. Int. J. Cancer.

[b29] Pierce JP, Patterson RE, Senger CM, Flatt SW, Caan BJ, Natarajan L (2014). Lifetime cigarette smoking and breast cancer prognosis in the After Breast Cancer Pooling Project. J. Natl Cancer Inst.

[b30] Fentiman IS, Allen DS, Hamed H (2005). Smoking and prognosis in women with breast cancer. Int. J. Clin. Pract.

[b31] Sagiv SK, Gaudet MM, Eng SM, Abrahamson PE, Shantakumar S, Teitelbaum SL (2007). Active and passive cigarette smoke and breast cancer survival. Ann. Epidemiol.

[b32] Dal Maso L, Zucchetto A, Talamini R, Serraino D, Stocco CF, Vercelli M (2008). Effect of obesity and other lifestyle factors on mortality in women with breast cancer. Int. J. Cancer.

[b33] Berube S, Lemieux J, Moore L, Maunsell E, Brisson J (2014). Smoking at time of diagnosis and breast cancer-specific survival: new findings and systematic review with meta-analysis. Breast Cancer Res.

[b34] Carey LA, Perou CM, Livasy CA, Dressler LG, Cowan D, Conway K (2006). Race, breast cancer subtypes, and survival in the Carolina Breast Cancer Study. JAMA.

[b35] Zhu BQ, Heeschen C, Sievers RE, Karliner JS, Parmley WW, Glantz SA (2003). Second hand smoke stimulates tumor angiogenesis and growth. Cancer Cell.

[b36] Abramowitz MC, Li T, Morrow M, Anderson PR, Bleicher RJ, Goldstein LJ (2010). History of smoking is associated with younger age at diagnosis of breast cancer. Breast J.

[b37] Manjer J, Malina J, Berglund G, Bondeson L, Garne JP, Janzon L (2001). Smoking associated with hormone receptor negative breast cancer. Int. J. Cancer.

[b38] Lilla C, Ambrosone CB, Kropp S, Helmbold I, Schmezer P, von Fournier D (2007). Predictive factors for late normal tissue complications following radiotherapy for breast cancer. Breast Cancer Res. Treat.

[b39] Pierce JP, Messer K, White MM, Cowling DW, Thomas DP (2011). Prevalence of heavy smoking in California and the United States 1965-2007. JAMA.

[b40] Peto J (2012). That the effects of smoking should be measured in pack-years: misconceptions 4. Br. J. Cancer.

