# Supplementary material for: Smoking and mortality after breast cancer diagnosis: the health and functioning in women study
Source: Cancer Med. 2014 Dec 16;4(2):315–24. doi: 10.1002/cam4.359 (PMC4329014; doi:10.1002/cam4.359)
Supplement: Supplementary file 1 [file cam40004-0315-sd1.docx]

**Table S1.** Hazard ratios (and 95% confidence intervals) of smoking intensity for mortality

|  |  | **Other Cause Mortality (No. deaths = 436)** | | |  |  | **Breast Cancer Mortality (No. deaths = 317)** | |  |
| --- | --- | --- | --- | --- | --- | --- | --- | --- | --- |
| **Exposure** | **N** | **Deaths** | **Unadjusted** | **Multivariate Adjusted** |  | **Deaths** | **Unadjusted** | **Multivariate Adjusted** |  |
| **Smoking status and intensity** |  |  |  |  |  |  |  |  |  |
| Never smoker | 494 | 238 | 1 | 1 |  | 151 | 1 | 1 |  |
| Former smoker, <20 py* | 154 | 52 | 1.10 (0.79, 1.52) | 1.17 (0.84, 1.63) |  | 50 | 1.02 (0.73, 1.43) | 0.88 (0.62, 1.26) |  |
| Former smoker, ≥20 py | 132 | 60 | 1.90 (1.38, 2.62) | 1.90 (1.37, 2.64) |  | 44 | 1.12 (0.79, 1.59) | 1.01 (0.70, 1.47) |  |
| Current Smoker, <20 py | 63 | 27 | 2.34 (1.49, 3.67) | 2.26 (1.42, 3.60) |  | 19 | 1.06 (0.64, 1.76) | 1.02 (0.60, 1.73) |  |
| Current smoker, ≥20 py | 132 | 59 | 2.62 (1.87, 3.67) | 2.58 (1.82, 3.64) |  | 53 | 1.46 (1.04, 2.04) | 1.57 (1.10, 2.25) |  |
|  |  |  |  |  |  |  |  |  |  |

*py: pack-years.

Unadjusted models were stratified by age at breast cancer diagnosis. Multivariate models were additionally adjusted for breast cancer treatment, race/ethnicity, financial adequacy, education, positive lymph node involvement, tumor size at diagnosis, comorbidity, and period of study entry.
